# *FNDC3B* promotes cell migration and tumor metastasis in hepatocellular carcinoma

**DOI:** 10.18632/oncotarget.10374

**Published:** 2016-07-01

**Authors:** Chin-Hui Lin, Yao-Wen Lin, Ying-Chun Chen, Chen-Chung Liao, Yuh-Shan Jou, Ming-Ta Hsu, Chian-Feng Chen

**Affiliations:** ^1^ VYM Genome Research Center, National Yang-Ming University, Taipei, Taiwan; ^2^ Proteomics Research Center, National Yang-Ming University, Taipei, Taiwan; ^3^ Institutes of Biomedical Sciences, Academia Sinica, Taipei, Taiwan

**Keywords:** FNDC3B, hepatocellular carcinoma, metastasis

## Abstract

Recurrence and metastasis are common in hepatocellular carcinoma (HCC) and correlate with poor prognosis. We investigated the role of fibronectin type III domain containing 3B (*FNDC3B*) in HCC metastasis. Overexpression of *FNDC3B* in HCC cell lines enhanced cell migration and invasion. On the other hand, knockdown of *FNDC3B* using short-hairpin RNA reduced tumor nodule formation in both intra- and extra-hepatic metastasis. High levels of *FNDC3B* were observed in metastatic HCCs and correlated with poor patient survival and shorter recurrence time. Mutagenesis and LC-MS/MS analyses showed that FNDC3B promotes cell migration by cooperating with annexin A2 (ANXA2). Furthermore, *FNDC3B* and *ANXA2* expression correlated negatively with patient survival. Our results indicate that *FNDC3B* behaves like an oncogene by promoting cell migration. This suggests *FNDC3B* could serve as a biomarker and therapeutic target for HCC metastasis.

## INTRODUCTION

Hepatocellular carcinoma (HCC) is one of the most common and aggressive tumors worldwide [1, 2]. In spite of therapeutic advancements that can extend the lifespan of patients [2], HCC is still the third leading cause of cancer-related deaths in the world [3]. HCC has a high frequency of recurrence after routine surgical treatment [4], and metastasis to the lungs, lymph nodes, bones, and adrenal glands is common [5, 6].

A genome-wide approach to identify common copy number alternation regions in the genome, targeting either amplifications or deletions, can reveal novel cancer genes. In a previous study, we screened for genomic aberrations in HCC cell lines and identified a 329 kb amplicon in 3q26.3 containing only 1 gene, fibronectin type III domain containing 3B (*FNDC3B*), which was upregulated in HCC tissues and cell lines [7]. In addition, the knockdown of *FNDC3B* decreased anchorage independent growth (AIG) and tumor formation in xenograft models [7].

*FNDC3B*, also called *FAD104* (factor for adipocyte differentiation 104), is a known regulator of adipocyte and osteoblast differentiation [8–10]. The fibronectin type III (FNIII) domains of the FNDC3B protein are involved in cell adhesion and growth signaling [11, 12]. Cell adhesion is important during tumorigenesis as it provides tumor cells with the necessary cell-to-cell contacts and cell-matrix interactions for cell signaling, proliferation, and migration [13]. In addition, the homozygous disruption of *FNDC3B* causes rapid postnatal death in mice and has pronounced effects on the adhesion, spreading, and migration of mouse embryo fibroblasts [9]. Therefore, we hypothesized that *FNDC3B* promotes cell migration and tumorigenesis in HCC. In this study, we investigated the role of *FNDC3B* in HCC through *in vitro* and *in vivo* experiments to understand its mode of action.

## RESULTS

### *FNDC3B* promoted cell migration and invasion in HCC cell lines

**Figure 1:**
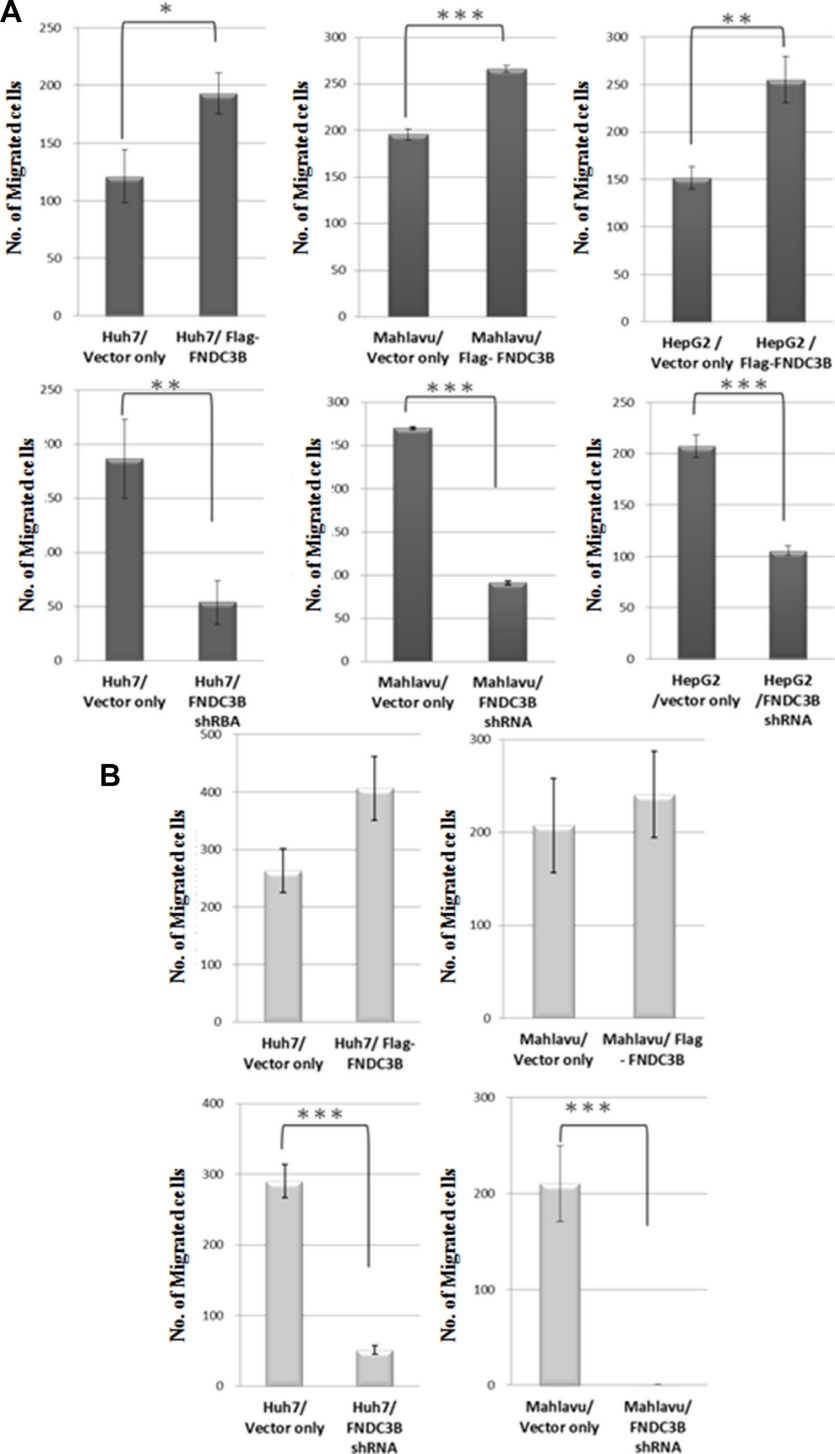
*FNDC3B* enhanced cell migration and invasion (**A**) Migration assay for overexpressed or knockdown cells utilized a transwell system. (**B**) Cell invasion assays (similar to the migration assays) with collagen pre-coated inserts (10 μg/cm^2^). **and*** represent *p* < 0.005 and *p* < 0.0005, respectively.

FNDC3B was commonly expressed in HCC cell lines (Figure S1A). To study whether FNDC3B indeed promotes cell migration and invasion, full-length expression vector or shRNA for *FNDC3B* was transfected into HCC cell lines. Knockdown or overexpression of FNDC3B does not significantly alter the cell growth rate (Figure S1B). As shown in Figure 1A, overexpression of *FNDC3B* enhanced migration in most of the HCC cell lines test as compared with vector only. Interestingly, knocking down the expression of FNDC3B resulted in inhibition of cell migration and invasion (Figure 1B). In Mahlavu and Huh7 cells, migration and invasion decreased more than 30% when *FNDC3B* was knocked down. This suggests that *FNDC3B* promotes cell migration and invasion during tumorigenesis.

### *FNDC3B* knockdown in highly invasive Mahlavu cells reduced tumor nodule formation *in vivo*

**Figure 2:**
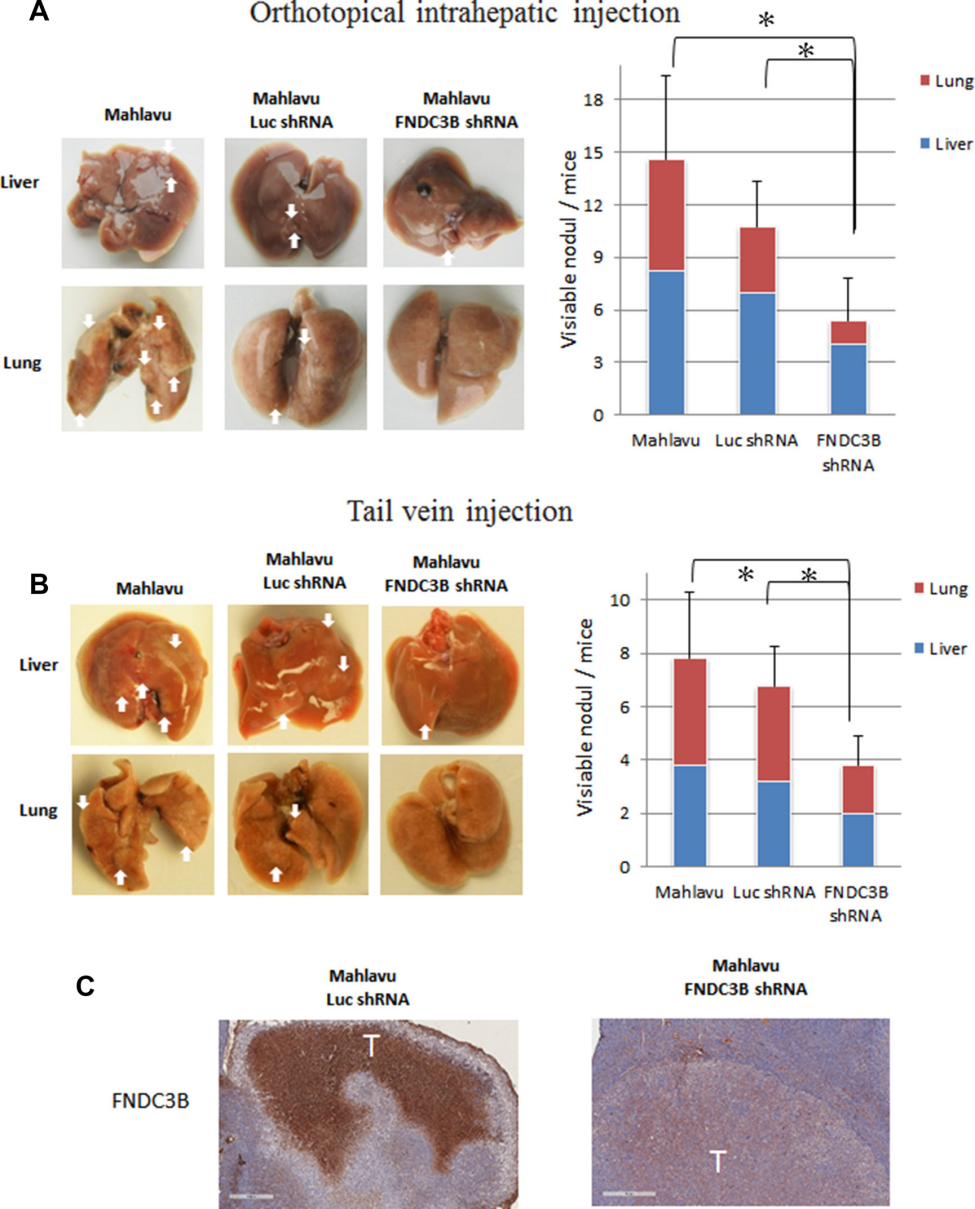
Knockdown of endogenous *FNDC3B* expression suppressed tumor metastasis in nude mice model Metastatic nodules in nude mice by (**A**) orthotopical intrahepatic or (**B**) tail-vein injection of Mahlavu cells. The white arrows indicate metastatic tumors. The metastatic number in liver and lung was calculated in nude mice. *represents *p* < 0.05. (**C**) Immunohistochemical staining of FNDC3B in liver tumor model section. Scale bar = 400 μm.

To validate our *in vitro* observations and investigate whether *FNDC3B* could serve as therapeutic target for metastatic HCC, we established a stable cell line of Mahlavu cells with knockdown of *FNDC3B*. The stable knockdown cells or the control Mahlavu cells were mixed with matrigel and injected into nude mice by orthotopic intrahepatic or tail vein injection. Six weeks after injection, the mice were sacrificed and visible tumor nodules were observed on the surface of the liver and lungs. The mice receiving injections of *FNDC3B* knockdown cells produced less than 60% tumor nodules compared with the vector control and original cells either by orthotopic intrahepatic (Figure 2A) or tail vein injection (Figure 2B) (*p* = 0.0216 and *p* = 0.0102, respectively). Immunohistochemical analysis of tumor nodule sections indicated the tumor nodule expressed human FNDC3B (Figure 2C). Consistent with our *in vitro* results, these results indicated that knockdown of *FNDC3B* significantly inhibits metastasis in HCC.

### FNDC3B overexpression correlates positively with HCC metastasis and negatively with patient survival

**Figure 3:**
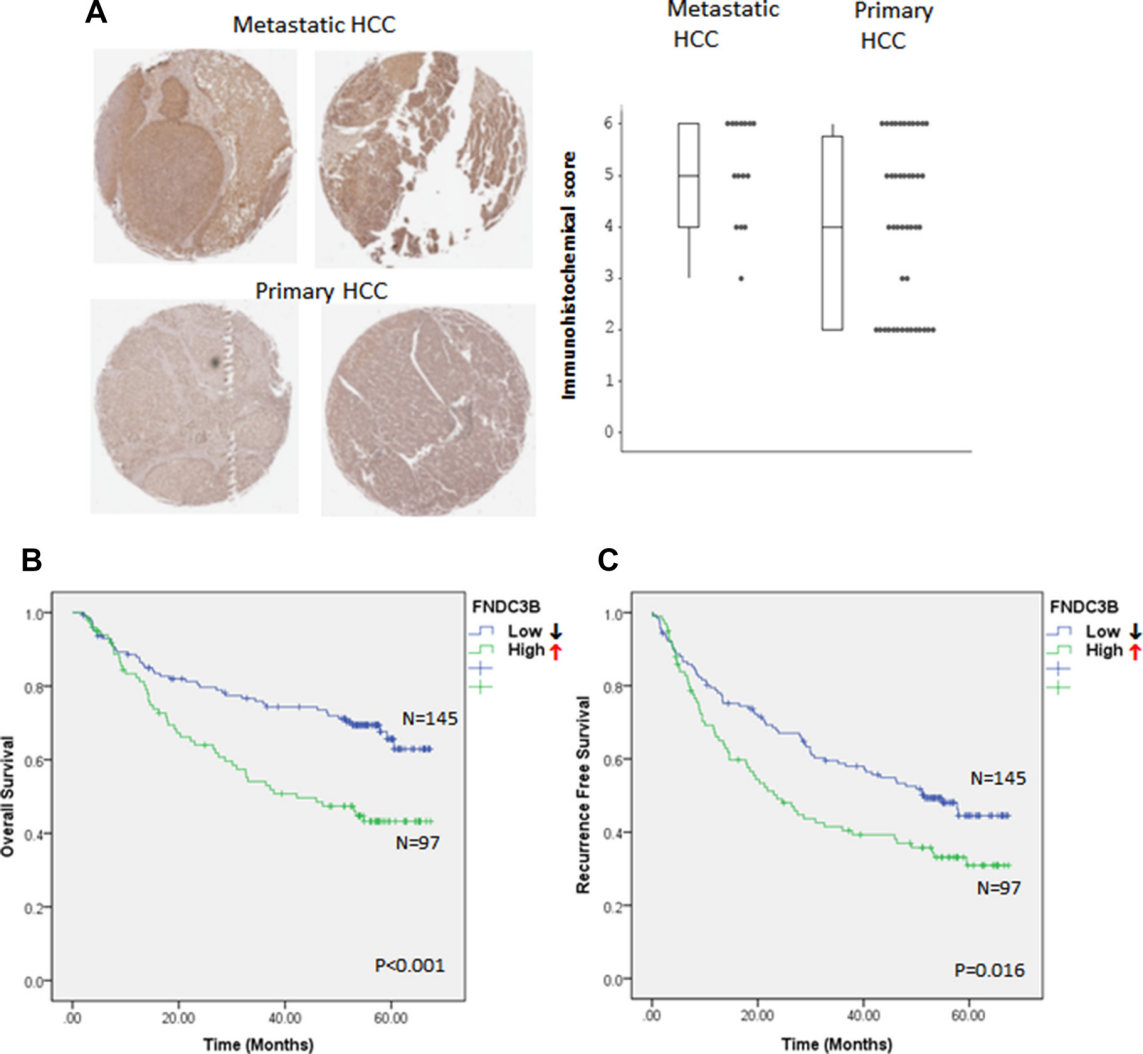
*FNDC3B* overexpression correlates with HCC metastasis and patient survival (**A**) FNDC3B levels in tissue arrays detected by FNDC3B antibodies in metastatic and primary HCC. (**B**) Differential immunohistochemical (IHC) score of FNDC3B between metastatic and primary HCC. (**C**) Overall survival and (**D**) recurrence-free survival of HCC patients categorized accordingly to the expression of *FNDC3B*. ↑ and ↓ stand for high (T/N ≥ 1.5) and low (T/N < 1.5) expression groups, respectively.

Both *in vitro* and *in vivo* studies suggested that the expression of *FNDC3B* correlates positively with metastasis in HCC. To further confirm the role of *FNDC3B* in tumor metastasis, we analyzed the expression of *FNDC3B* in 15 metastatic and 44 primary HCC tissues by tissue array (Figure S2). Interestingly, *FNDC3B* was overexpressed in metastatic HCC tissues (73.33%, 11/15) compared with primary HCCs (45.45%, 20/44) (*p* = 0.003) (Figure 3A–3B). To further validate our observations, we analyzed 242 HCC microarray data obtained from public databases. Kaplan-Meier analysis showed that upregulation of *FNDC3B* correlated negatively with patient survival (*p* < 0.001) (Figure 3C). The average survival time was 41.22 months for HCC patients with upregulated *FNDC3B* and 52.37 months for patients without *FNDC3B* upregulation. Patients with upregulated *FNDC3B* presented a 64.65% recurrence rate and an average of 33.4 months recurrence time, while HCC patients without *FNDC3B* upregulation had a recurrence rate of 51.1% and average recurrence time of 42.53 months (*p* = 0.016) (Figure 3D). Our survival analysis suggests the upregulation of *FNDC3B* correlates positively with recurrence and negatively with patient survival.

### FNIII domains 1–4 of FNDC3B were essential for cell migration

**Figure 4:**
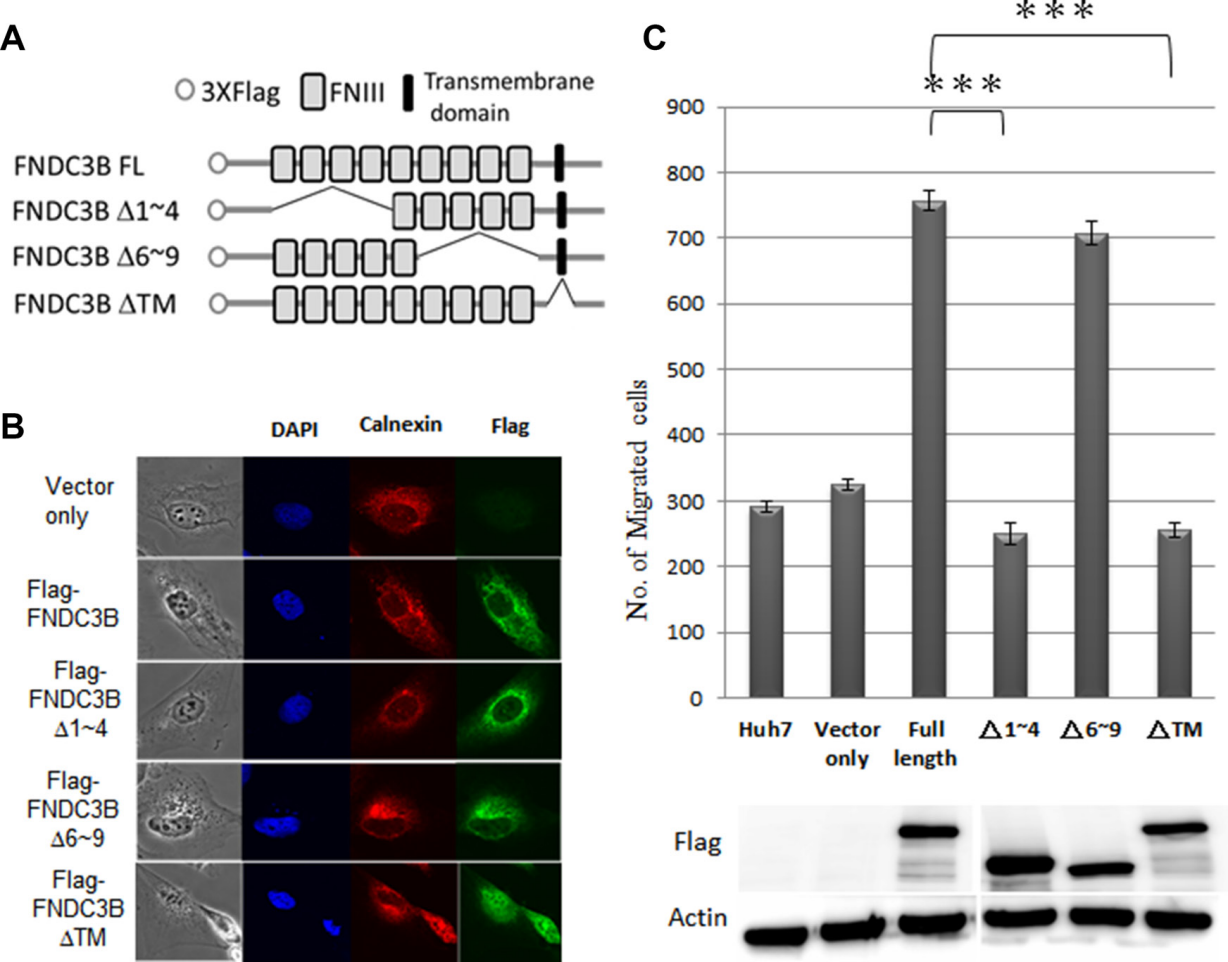
FNIII domains 1–4 were essential for *FNDC3B* to enhance cell migration and invasion (**A**) The construction map of variant FNIII domain deletion mutants of FNDC3B. (**B**) Cellular localization of full-length *FNDC3B* and variant domain deletion mutants assessed by immunofluorescence. Calnexin and Flag-FNDC3B visualized with TRITC-conjugated calnexin (red) and anti-Flag antibody (green), respectively. (**C**) Migration assays for FNIII domain deletion mutants performed using the transwell system. ***represent *p* < 0.0005.

FNDC3B is composed of 9 FNIII domains and a transmembrane domain (Figure 4A). FNIII has been shown to serve as a scaffold for the generation of stable protein domains that bind to variable proteins [14, 15]. To explore the role of FNIII domains in cell migration, we constructed partial FNIII deletion mutants of FNDC3B and analyzed their migration ability (Figure 4B–4C; Figure S3). The cellular distribution of flag-FNDC3B overlapped with the staining pattern of calnexin, a marker for the endoplasmic reticulum (ER), suggesting that FNDC3B localizes to the ER membrane (Figure 4B). The transmembrane domain deletion mutants redirected their cellular localization to the nucleus and lost the ability to promote migration (Figure 4B–4C). Therefore, cellular localization of FNDC3B on the ER membrane is important for the FNDC3B's migration-inducing activity. Of note, we found that the Δ6~9 mutants exhibited similar migration capabilities as the full-length construct, but the Δ1~4 mutants showed no difference compared with the vector only control (Figure 4C; Figure S3). These results suggest that the first four FNIII domains are responsible for FNDC3B's functions related to cell migration.

### FNDC3B cooperated with ANXA2 to promote cell migration

**Figure 5:**
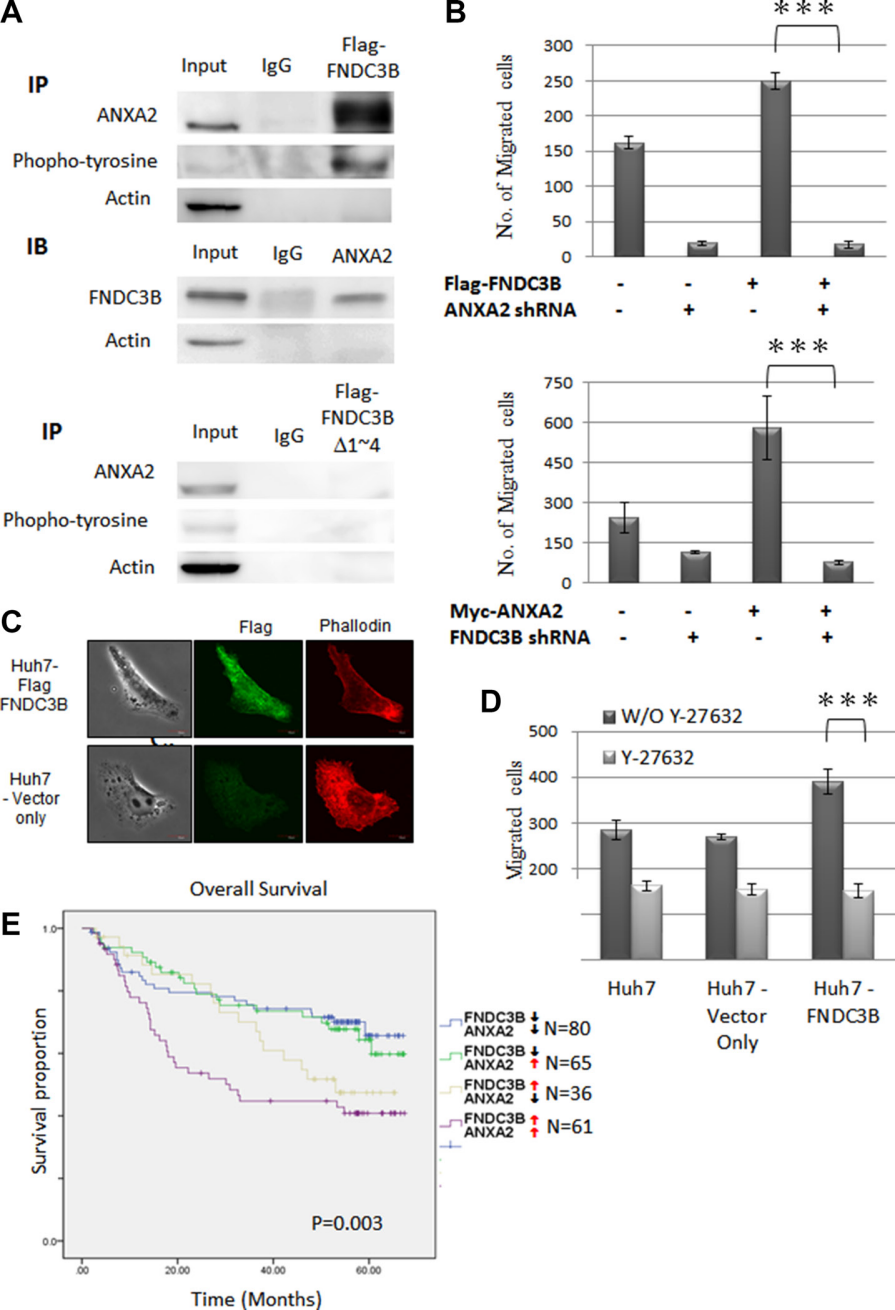
ANAX2 interacting FNIII domains 1–4 of FNDC3B were essential for *FNDC3B* enhanced cell migration (**A**) Co-immunoprecipitation of Flag-FNDC3B and ANAX2, using lysates from Huh7 cells transfected with Flag-FNDC3B for immunoprecipitation (IP) with anti-Flag. (**B**) Migration assay for *FNDC3B* overexpressed Huh7 cells treated with *ANXA2* shRNA and *ANXA2* overexpressed Huh7 cells treated with *FNDC3B* shRNA. (**C**) Vector only and *FNDC3B* overexpression Huh7 cells observed by confocal microscopy. F-actin and Flag-FNDC3B were visualized with TRITC-conjugated phalloidin (red) and anti-Flag antibody (green), respectively. (**D**) Migration assay for *FNDC3B* overexpressed Huh7 cells treated with 1 mM/mL Y27632. ***represents *p* < 0.005. (**E**) Overall survival curves of HCC patients categorized according to FNDC3B and *ANXA2* expression.↑ and ↓ stand for high (T/N ≥ 1.5) and low (T/N < 1.5) expression groups, respectively.

Co-immunoprecipitation (co-IP) was performed to identify proteins interacting with wild type FNDC3B and Δ1~4 mutant. Proteins differentially binding to wild type and the mutant FNDC3B were analyzed by Liquid Chromatograph Tandem Mass Spectrometry (LC-MS/MS). One of the candidates, ANXA2, interacted with full-length FNDC3B but not with the Δ1~4 mutant. *ANXA2* overexpression has been suggested to be an HCC marker [16, 17]. The tyrosine-phosphorylated ANXA2 has also been shown to be involved in cell migration and transformation [18, 19]. In our immunoprecipitation experiments the full-length FNDC3B interacted with phosphorylated ANXA2 (Figured 5A). Cell migration assays indicated that *FNDC3B* overexpression induced cell migration, which was reversed by knocking down *ANXA2* (Figure 5B). The same results were observed when *ANXA2* was overexpressed in *FNDC3B* knockdown cells (Figure 5B). In previous reports, phosphorylation of ANXA2 has been shown to regulate Rho-mediated actin rearrangement [20–22]. In our immunofluorescence staining experiments, cells overexpressing FNDC3B presented more well-defined stress fibers along the cellular length (Figure 5C). In addition, Rho inhibitor (Y-27623) treatment reduced migration in cells overexpressing *FNDC3B* (*p* < 0.005) (Figure 5D). These observations suggest that FNDC3B cooperated with ANXA2 to promote cell migration by mediating actin rearrangement. Furthermore, we performed Kaplan–Meier analyses to measure the correlation between *FNDC3B* or *ANXA2* expression and HCC patient survival. Interestingly, the upregulation of *ANXA2* did not alter patient survival when *FNDC3B* was expressed at normal levels (52.15 months survival time compared to 52.36 when *ANXA2* was expressed at normal levels). However, the upregulation of *ANXA2* decreased patient survival when *FNDC3B* was also upregulated (37.78 months survival time compared to 46.45 months when *ANXA2* was expressed at normal levels, *p* = 0.003) (Figure 5E).

## DISCUSSION

In this study, we performed *in vitro* cell migration and invasion assays, *in vivo* metastasis assays, tissue array analyses, and survival analyses of patients to study the role of *FNDC3B* in HCC metastasis. Knocking down *FNDC3B* expression prevented intra- and extra-hepatic metastasis in our metastasis mouse model. Clinical data also indicated that the expression of *FNDC3B* correlated positively with HCC metastasis and negatively with patient survival. Additionally, protein interaction, cell migration and survival analyses suggested that FNDC3B cooperated with ANXA2 to promote cell migration and invasion.

By analyzing microarray data from public domain databases, we previously showed that *FNDC3B* was upregulated in HCC samples infected with HBV or HCV [7]. Here, our tissue array data showed that *FNDC3B* was highly expressed in normal liver tissue infected with viral hepatitis (45%, 9/20) (Figure S4). This observation suggests that the expression of *FNDC3B* correlates with hepatic virus infection. Since hepatic virus infection is the major cause of HCC, *FNDC3B* would be important for the tumorigenesis of HCC.

FNDC3B is mainly composed of FNIII domains. Individual FNIII repeats have a high degree of structural homology, despite displaying only 20–40% identity in their amino acid sequences. They are composed of ~90 amino acids organized in 7 anti-parallel β strands and are involved in cell adhesion and growth signaling [23]. In this study, we constructed domain deletion mutants to identify the FNDC3B domains that are involved in cell migration. Our results indicated that FNIII domains 1~4 are important for cell migration. Moreover, the transmembrane domain deletion mutant redirected the localization of FNDC3B in cells from the ER to the nucleus and FNDC3B lost its ability to promote cell migration. These results suggest that FNDC3B's localization to the ER membrane is essential for activating the cell migration signaling pathway.

ANXA2 is a calcium-dependent phospholipid-binding protein, which can cap filamentous actin to regulate membrane-membrane and membrane-cytoskeletal interactions [24]. ANXA2 has been implicated in many cellular functions such as exocytosis, endocytosis, vesicle transport, ion channel regulation, immune response, cell–to-cell adhesions, and fibrinolysis [24, 25]. ANXA2 can be phosphorylated at Tyr23 by Src kinase [26] to regulate Rho mediated actin rearrangement and cell morphology alteration [20–22]. *ANXA2* is aberrantly expressed in many types of cancer, including HCC [27–30]. In addition, tyrosine-phosphorylated ANXA2 is involved in cell migration and malignant transformation [18]. We observed that FNDC3B interacts with phosphorylated ANXA2 through the first four FNIII domains, which are essential for cell migration. Immunofluorescence staining and Rho inhibitor (Y-27623) treatment suggested that FNDC3B might rearrange actin by cooperating with phosphorylated ANXA2. However, further studies are necessary to understand the underlying mechanism.

*FNDC3B* is one of the most commonly upregulated genes in cancerous tissues, regardless of cancer type and tumor origin [31]. Here, we showed that *FNDC3B* was overexpressed in the metastatic HCC tumors and that *FNDC3B* expression correlated negatively with HCC patient survival. We also performed Kaplan-Meier survival analyses using microarray data (http://www.kmplot.com) from thousands of breast and ovarian cancer patients. *P* values (*p* = 2 × 10^−15^ for breast cancer and *p* = 0.00057 for ovarian cancer) indicated that FNDC3B expression also correlated negatively with patient survival in other cancers (Figure S5).

In a previous report, Zhang *et al*. found that miR-143 was highly expressed in a subset of metastatic HCC cases, positive for hepatitis B virus. Such study suggested that miR-143 promoted metastasis by inhibiting the expression of *FNDC3B* [32]. However, other evidence highlights *FNDC3B* as oncogene. First, *FNDC3B* is usually amplified [7, 33] and highly expressed in HCC and other cancers [7, 31, 34–36]. Cai *et al*. found that FNDC3B overexpression induces the epithelial-to-mesenchymal transition and activates several cancer pathways [33]. In addition, direct evidence from our knockdown and overexpression experiments indicates that *FNDC3B* promotes cell migration and tumor metastasis in HCC. Finally, disease-free survival analysis of cancer patients indicated that overexpression of FNDC3B correlates with recurrence and metastasis.

Tumor metastasis occurs in 90% of all cancer related deaths [37]. Thus, studies aimed at understanding metastasis might find therapeutic applications to improve patient survival. Our findings here suggest that FNDC3B could serve as diagnostic marker and therapeutic target for HCC.

## MATERIALS AND METHODS

### Antibodies and cell culture

Polyclonal anti-FNDC3B and monoclonal anti-Flag M2 antibodies were purchased from Sigma. Monoclonal anti-ANXA2 antibody, Alexa Fluor-488 goat anti-mouse IgG and Alexa Fluor-488 goat anti-rabbit IgG were purchased from Invitrogen (Invitrogen, CA, USA). Monoclonal anti-beta-tubulin and polyclonal anti-GAPDH antibodies were purchased from Enogene (Enogene, Taiwan). Monoclonal anti-Myc (9E10) and monoclonal anti-phophotyrosine (4G10), were purchased from Millipore (Millipore Corporation, MA, USA). The polyclonal anti-calnexin antibody was purchased from GeneTex (GeneTex, CA, USA). HCC cell lines (HepG2, HuH7 and Mahlavu) were cultured in Dulbecco's modified Eagle's medium supplemented with 10% fetal bovine serum, 1% nonessential amino acids, and 1% penicillin/streptomycin (Invitrogen, Carlsbad, CA). Cells viability was determined by the cell proliferation assay every 24 hours for 4 days using the AlamarBlue reagent (AbD Serotec, UK).

### Small interfering RNA and lentiviral infection

Short hairpin RNAs (shRNAs) targeting *FNDC3B* and *ANXA2* in the RNAi Consortium shRNA library were ordered from the National RNAi Core Facility (Academia Sinica, Taiwan). We selected and mixed the 2 or more effective shRNAs to knock down the expression of *FNDC3B* (TRCN0000082774 and TRCN0000082776) and *ANXA2* (TRCN0000056144, TRCN0000056145, TRCN0000289381, and TRCN0000296322) by infection with 293T-produced lentivirus. For lentivirus production, the supernatant of the 293T culture was harvested at 24, 48 and 72 h after transfection with shRNA vectors. Targeted cells were then incubated with lentiviruses for 24 h with 8 μg/mL polybrene (Sigma-Aldrich, MO, USA).

### Cell migration and invasion assays

The migration assay was performed in 24-well transwell units with an 8 mm pore size polycarbonate membrane (BD Biosciences, CA, USA). Cells that were re-suspended in a serum-free medium were seeded into the upper chamber of the insert and then placed into the bottom chamber containing 10% fetal bovine serum as a chemoattractant. Cells were allowed to migrate for 24 h, followed by methanol fixation and Giemsa staining (MERCK, Germany). Cells that did not migrate to the apical side of membrane were removed with a cotton swab. Migrated cells were photographed with a phase-contrast microscope. For the invasion assay, the membrane filters were coated with collagen to form a continuous thin layer. All experiments were performed in triplicates. Independent Student's *t* tests were used to compare the continuous variables between the 2 groups.

### *In vivo* tumor metastasis

For tail vein injections, cells (5 × 10^6^/mouse) were intravenously injected into athymic BALB/c nude (nu/nu) mice at 4 weeks of age. The mice were sacrificed 8 weeks after injection. The livers and lungs from the sacrificed mice were harvested and fixed in 10% formalin followed by 75% ethanol. The number of metastatic tumors was then assessed. For orthotopical injections, cells (5 ×10^6^/ mouse) were mixed with 50 μl matrigel and injected into the left lobes of the livers of the athymic BALB/c nude (nu/nu) mice at 4 weeks of age. The animals were sacrificed 6 weeks after injection and the number of metastatic tumors was assessed.

### Immunohistochemistry and scoring

Tissue microarrays (CS3 and CSN3) were purchased from SuperBioChips Labs (SuperBioChips Labs, Korea). The immunohistochemical methodology followed the standard protocol provided by the manufacturer's instructions. Immunohistochemical staining was scored according to the intensity of staining (no staining = 0, weak staining = 1, moderate staining = 2, strong staining = 3) and the extent of the cell stained (0% = 0, 1–10% = 1, 11–50% = 2, > 50% = 3). The final immunoreactive score was determined by adding the intensity scores with the extent of positivity scores of stained cells. Immunohistochemical analyses and scoring were performed by 3 independent investigators.

### Microarray analysis and kaplan-meier survival analysis

For analysis of *FNDC3B* expression in HCCs, gene expression microarray datasets were downloaded from the public domain GEO database (GSE14520). The gene expression profiling experiments were performed using Affymetrix HG U133 series arrays. Since the scanned images of each array may have different overall brightness, the dChip (www.dchip.org) software was applied to normalize the brightness of the arrays to comparable levels. The tumor and comparative normal tissue arrays were entered into the dChip. The array corresponding to the median overall intensity was used as the baseline array to adjust the overall probe intensity level for all other arrays. Then the intensity of *FNDC3B* normalized probe sets were output for analysis. *FNDC3B* upregulation was defined as greater than 1.5 fold increase above the average intensity of 7 normal livers. Kaplan–Meier survival analysis was conducted using SPSS v.20 (IBM, USA).

### In-gel digestion and mass spectrometric analysis

Distinct protein bands were manually picked from the SDS-PAGE and unstained in a solution of 25 mM NH_4_HCO_3_ and 50% (v/v) acetonitrile (1:1). The gel pieces were dehydrated in acetonitrile for 10 min, vacuum dried, and rehydrated in 2% (v/v) β-mercapteoethanol for 20 min, followed by alkylation in 25 mM ammonium bicarbonate containing 5% 4-vinylpyridine, 50% acetonitrile for 20 min. Then gel pieces were washed twice with ammonium bicarbonate (25 mM) at room temperature. Subsequently, the gels were vacuum dried, and finally digested with 25 ng trypsin (Promega, WI, USA) in 25 mM ammonium bicarbonate (pH 8.5) at 37 °C overnight. The tryptic peptides were extracted from gels and dried by Speed-Vac (Thermo Electron, MA, USA). Each tryptic digest was dissolved in 0.1% formic acid and loaded into an LTQ-Orbitrap Discovery hybrid mass spectrometer with a nanoelectrospray ionization source (ThermoElcetron, CA, USA) coupled to a nano-flow HPLC (Agilent Technologies 1200 series). Mobile phase solvent A and B were prepared as 0.1% formic acid in water and 0.1% formic acid in acetonitrile. Peptides were separated in a tip column (13.5 cm length, 75 μm inner diameter, 5 μm beads, YMC-Gel, Lipid Chromatography) with a linear gradient of 3%–40% B for 90 min, 40%–95% B for 2 min, 95% B for 10 min at a flow rate of 0.5 μl/min. Eluted peptides were ionized by a spray voltage of 2.3 kV and introduced into the mass spectrometer. Mass spectrometric data were obtained using a data-dependent acquisition method, in which one full MS survey scan (m/z: 200–2000) was set at a high resolution of 30,000 (full-width at half-maximum) followed by scanning for the first six most highly charged ions (2+ and 3+). Fragmented peptide ions of each selected precursor peptide ion were generated by collision-induced dissociation (CID) using helium gas with 35% collision energy.

### Protein database search

The TurboSequest search server (ver. 27, rev. 11; Thermo Electron, Waltham, MA, USA) was used to identify the peptide sequences against a UniProt human protein database (containing 127,738 protein sequences; released on February, 2015; http://www.uniprot.org/). A protein was identified when more than two peptides with an Xcorr score higher than 2.5 (for doubly charged ions) or 3.75 (for triply charged ions) were matched.

## SUPPLEMENTARY MATERIALS FIGURES AND TABLES



## Abbreviations

HCC: hepatocellular carcinoma
FNDC3B: fibronectin type III domain containing 3B
ANXA2: annexin A2
FNIII: fibronectin type III domain
LC-MS/MS: Liquid Chromatograph Tandem Mass Spectrometer.
