# Supplementary material for: FNDC3B promotes cell migration and tumor metastasis in hepatocellular carcinoma
Source: Oncotarget. 2016 Jul 1;7(31):49498–508. doi: 10.18632/oncotarget.10374 (PMC5226524; doi:10.18632/oncotarget.10374)
Supplement: Supplementary file 1 [file oncotarget-07-49498-s001.pdf]

FNDC3B promotes cell migration and tumor metastasis in hepatocellular carcinoma

Supplementary Materials

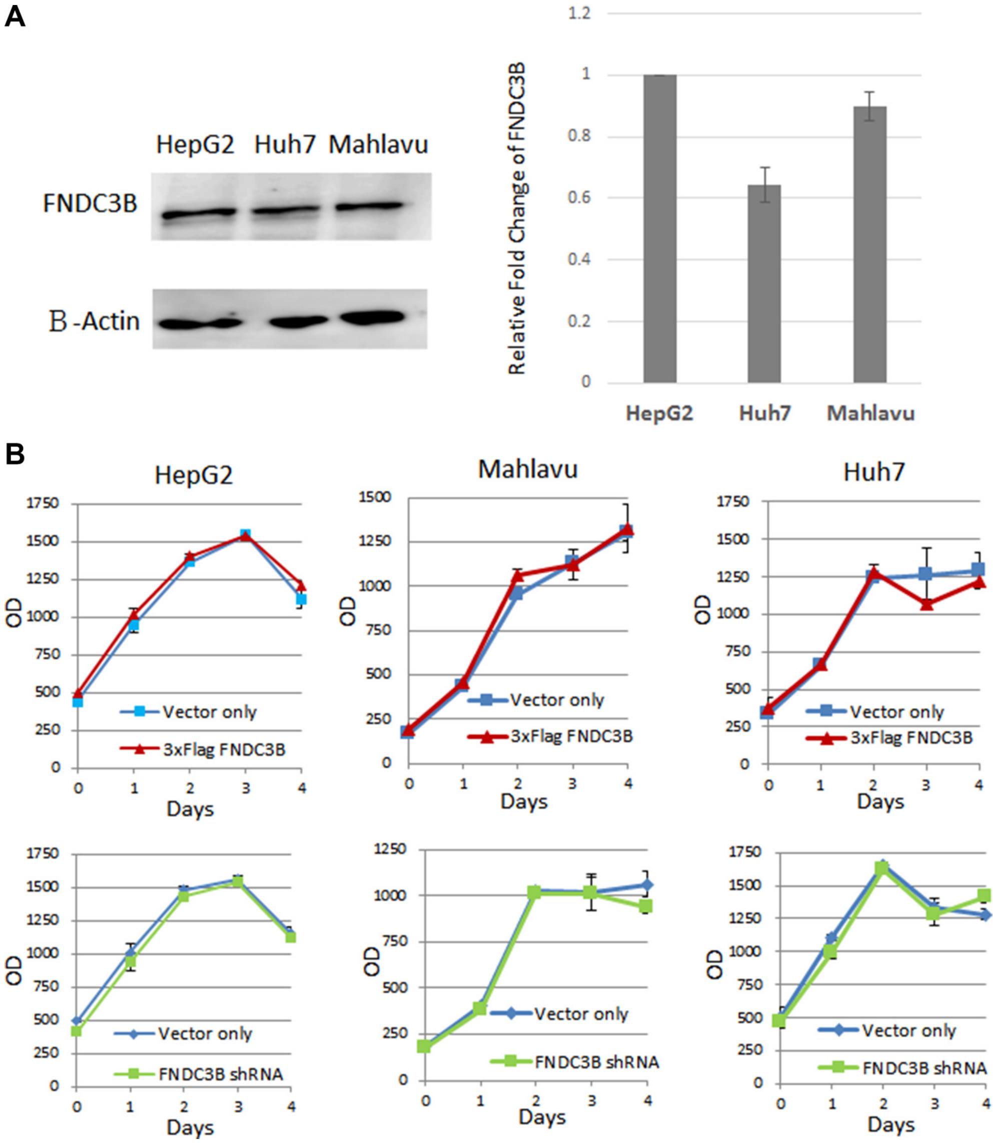

Supplementary Figure S1: (A) Western blot and QRT-PCR for FNDC3B in HCC cell lines. (**B**) Proliferation assay for the FNDC3B overexpressed and knockdown HCC cell lines.

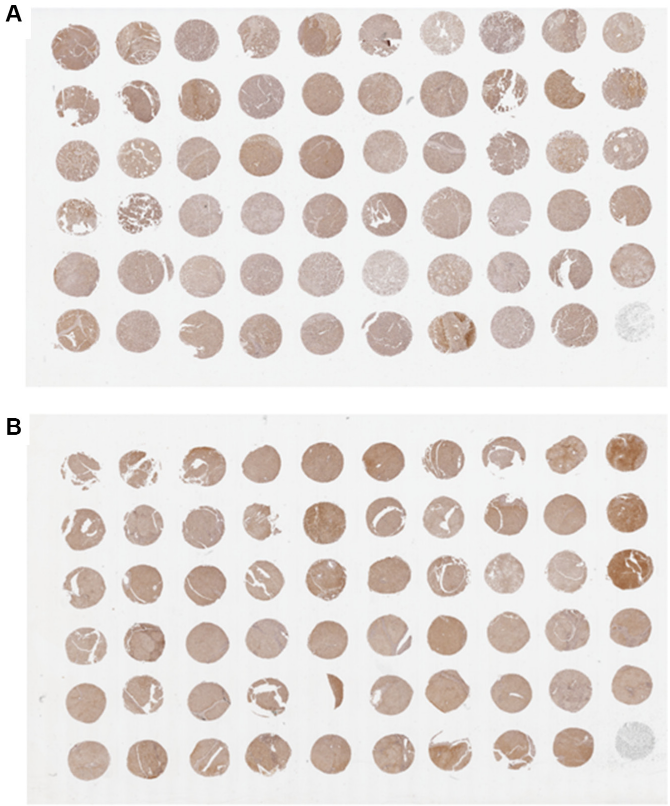

Supplementary Figure S2: Immunohistochemistry analysis of the expression of FNDC3B in HCC (A) and normal liver (B) tissue microarrays.

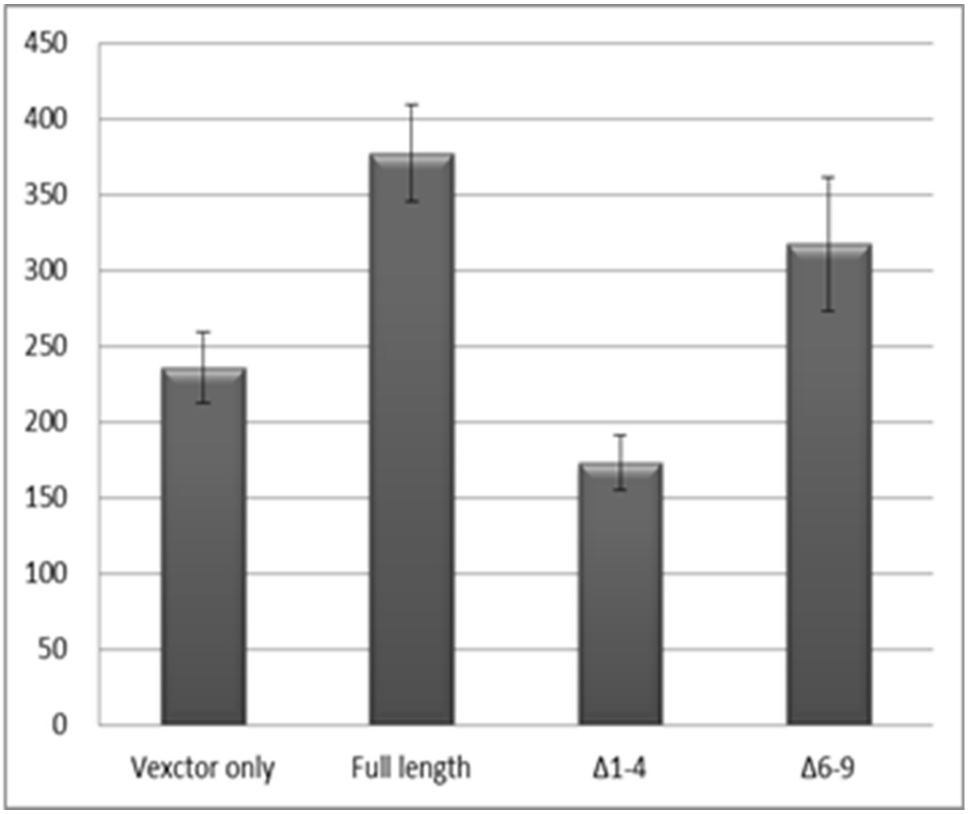

Supplementary Figure S3: Migration assay for the FNIII domain deletion mutants in Tong cells were performed using the transwell system.

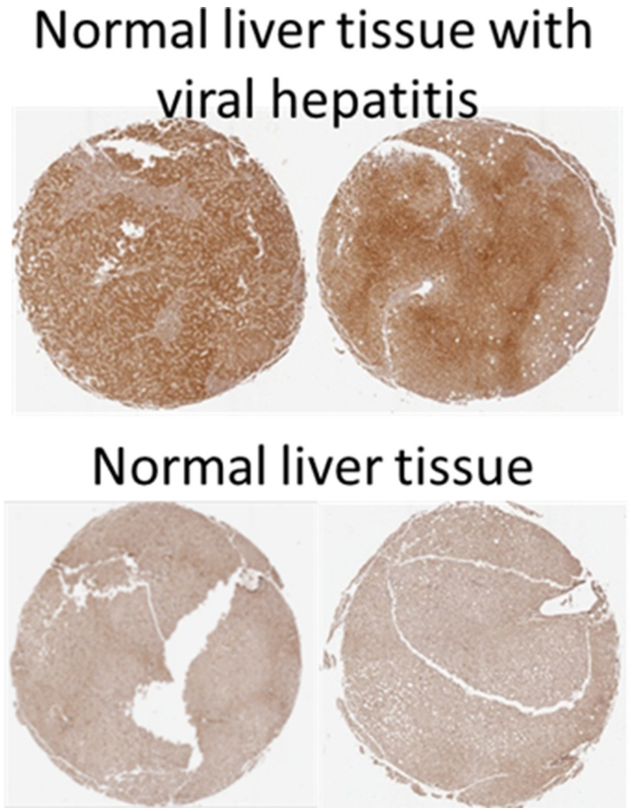

Supplementary Figure S4: FNDC3B levels in the tissue array were detected by FNDC3B antibodies in Normal liver.

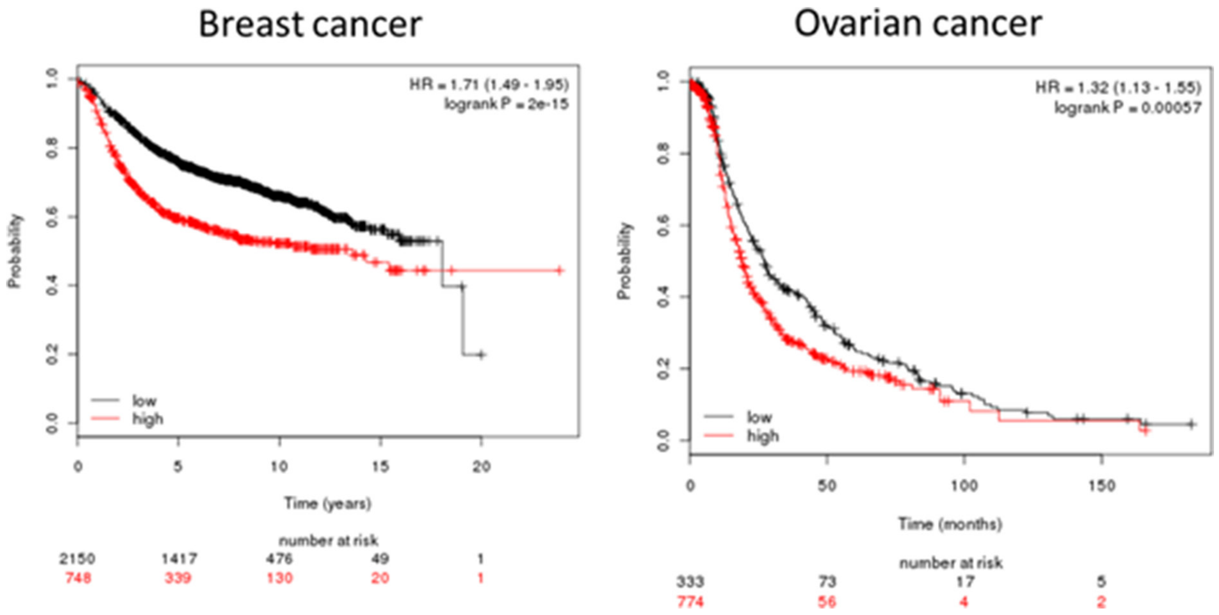

Supplementary Figure S5: Overall survival curves of breast and ovarian cancer patients categorized accordingly to the expression of FNDC3B.
